# SatHealth: A Multimodal Public Health Dataset with Satellite-based Environmental Factors

**DOI:** 10.1145/3711896.3737440

**Published:** 2025-08-03

**Authors:** Yuanlong Wang, Pengqi Wang, Changchang Yin, Ping Zhang

**Affiliations:** The Ohio State University, Columbus, Ohio, USA; The Ohio State University, Columbus, Ohio, USA; The Ohio State University, Columbus, Ohio, USA; The Ohio State University, Columbus, Ohio, USA

**Keywords:** Satellite Images, Environmental Health Informatics, Social Determinants of Health, Medical Records, AI in Public Health

## Abstract

Living environments play a vital role in the prevalence and progression of diseases, and understanding their impact on patient’s health status becomes increasingly crucial for developing AI models. However, due to the lack of long-term and fine-grained spatial and temporal data in public and population health studies, most existing studies fail to incorporate environmental data, limiting the models’ performance and real-world application. To address this shortage, we developed SatHealth, a novel dataset combining multimodal spatiotemporal data, including environmental data, satellite images, all-disease prevalences estimated from medical claims, and social determinants of health (SDoH) indicators. We conducted experiments under two use cases with SatHealth: regional public health modeling and personal disease risk prediction. Experimental results show that living environmental information can significantly improve AI models’ performance and temporal-spatial generalizability on various tasks. Finally, we deploy a web-based application^1^ to provide an exploration tool for SatHealth and one-click access to both our data and regional environmental embedding to facilitate plug-and-play utilization. SatHealth is now published with data in Ohio, and we will keep updating SatHealth to cover the other parts of the US. With the web application and published code pipeline^2^, our work provides valuable angles and resources to include environmental data in healthcare research and establishes a foundational framework for future research in environmental health informatics.

## 1 Introduction

The living environment (*e.g*., climate, green spaces, air quality, and socioeconomic factors) considerably impacts people’s physical [20, 25] and mental [67] health, and understanding such impact is an emerging topic [18, 32, 38]. Considerable efforts have been made to explore how these factors are associated with human health [36, 60, 63]. However, due to the complexity of aligning multi-source geospatial data with medical data, there are still limited handy medical datasets equipped with high spatiotemporal resolution, long-term coverage, and comprehensive environmental data. Therefore, existing studies either focus on specific diseases and environmental factors [14, 40] or take solely patients’ medical record histories without consideration of environmental factors [13, 69, 74, 75]. As a result, these models might be limited in their accuracy, comprehensiveness, and spatiotemporal robustness. Moreover, the benefit of information from the living environment in healthcare AI remains unexplored.

To fill the shortage, we propose a pipeline and develop SatHealth, a compound dataset featuring satellite-based environmental data, satellite imagery, prevalence of all diseases estimated from medical claims, and Social Determinants of Health (SDoH) indices. To the best of our knowledge, SatHealth is the first dataset in the US that combines regional environmental characteristics with a healthcare database. We requested over 400k aerial view satellite images from Google Maps [27], each image covers about a 500m wide square area. Furthermore, we use medical claims from the MarketScan [44] database to estimate the regional prevalence for all diseases. As for SDoH, we use the Social Deprivation Index (SDI) [33], which is a comprehensive score calculated from US census data from the American Community Survey (ACS) [9]. Moreover, we designed a multimodal fusion framework to seamlessly integrate heterogeneous multimodal environmental data sources and provide user-friendly regional environment embeddings, facilitating downstream analyses and follow-up studies.

We first validated and quantified the environmental-disease relationship by statistical testing, reflecting regional health status disparity. After that, we use the dataset for two clinical tasks: regional public health modeling (*e.g*., to predict regional SDI scores and disease prevalence based on environmental data) and personalized disease risk prediction (*e.g*., to enhance personal disease risk prediction with environmental data). The experimental results show that living environmental information can significantly improve AI models’ performance and spatiotemporal generalizability. Finally, we deployed a web-based application^1^ where users could explore and access SatHealth data with regional embedding vectors. Our regional embeddings can be plugged into any clinical AI with geospatial information, which paves the way for integrating environmental factors into clinical AI development.

We started SatHealth development from Ohio as a concomitant of the Ohio O-SUDDEn program [23]. However, all satellite data we use have global coverage, and MarketScan patient-level medical claims have US coverage. Therefore, our framework’s environmental factor processing pipeline can be easily adapted to other areas. We also provide our code on GitHub^2^ so that users can create data and embeddings for different areas of interest. We will also gradually update SatHealth to cover the US in the future.

In summary, we summarize our contributions as follows:

- We construct SatHealth, the first publicly available dataset in the US consisting of environmental data, satellite imagery, regional SDoH, and all-disease prevalence for comprehensive environment-health analysis.
- We design an embedding pipeline to construct regional environment embeddings by fusing multimodal data from SatHealth.
- We show two use cases of SatHealth. The experimental results exhibit the benefit of environmental information in model accuracy and temporal-spatial generalizability.
- We deployed a web-based application to showcase and provide access to SatHealth and our embeddings. We also publish our code for data collection in other areas of interest.

**Code and Docs**: https://github.com/Wang-Yuanlong/SatHealth **Web app and Dataset download**: https://aimed-sathealth.net **License**: The dataset is released under the CC BY-SA 4.0 license.

## 2 Related Works

### 2.1 Environment-health Datasets

Environmental factors, such as built environment, air quality, and green space, are reported to be correlated with many diseases, such as heart diseases [14, 15], metabolic syndrome [37], and stroke [4]. Researchers construct datasets with earth observation (EO) and remote sensing data with various health outcomes to learn their health impacts. For example, Barboza et al. [7] and Temenos et al. [61] collected regional greenery indices like normalized difference vegetation index (NDVI) and percentage of green area (%GA), and studied their relationship with natural-cause mortality and COVID-19 cases. However, the greenery indices provide a limited expression of the living environment. Therefore, some datasets include satellite images to capture a more comprehensive neighborhood view. SatelliteBench [47] examines the dengue outbreak in 81 Colombian municipalities, collecting Sentinel-2 satellite images, climate, socioeconomic factors, and dengue cases to predict poverty, school access, and dengue outbreaks. Moreover, SustainBench [71] benchmarks prediction tasks of 4 public health indicators from surveys with satellite images and street view panorama. However, These datasets rely on public health surveys, which limit their target scope. Recently, MedSat [56] was developed in England by integrating sociodemographic features, satellite imagery, environmental variables, and prescription data. However, it is constrained by indirect prevalence estimation, leading to higher errors and limited disease scope. Finally, all these datasets suffered from a limited scope of target disease and none or moderate-resolution satellite images. Inspired by all these works, we developed SatHealth by combining SDoH, environmental variables, satellite images, and the MarketScan medical claim database. High-resolution satellite images from Google Maps provide a clear view of ground-level environments, while MarketScan provides prevalence estimates for thousands of diseases. Hence, SatHealth enables comprehensive environment-health analyses.

### 2.2 Health-related Target Modeling

#### Environment-health relationship modeling.

Numerous studies have been conducted to explore the impact of the environment on human health. The fundamental methods are community surveys and statistical tests. Wafula et al. [63] used surveys and mediation analysis to explore socioeconomic disparities in malaria prevalence in Malawi. Similarly, Keenan et al. [36] analyzed multidrug-resistant urinary tract infections (MDR UTIs) in East Africa using questionnaires and Bayesian profile regression to identify clusters of social and environmental determinants. By incorporating image modalities, Zhang et al. [76] explored the protective effects of shaded environments against adolescent myopia using commercial satellite maps and Spearman’s correlation analysis.

Machine learning methods are also widely adopted. For example, Araújo et al. [3] employed Sentinel-1 and Sentinel-2 data to investigate the relationship between green spaces and mental health, using spatial autocorrelation metrics like Moran’s I and regression models to quantify the impact of environmental features. Yin et al. [73] and Nazia et al. [49] applied Bayesian hierarchical models to study the dynamics of COVID-19 spread, incorporating environmental, social, and health data. Luo et al. [40] used mixed-effects models to analyze the association between air pollution and hypertension, revealing disparities in cardiovascular health risks. At the same time, Gibb et al. [26] employed Bayesian spatiotemporal modeling to investigate dengue emergence linked to climate change and urban infrastructure. Most recently, Chen et al. [14] investigated the correlation between built environment from street view and coronary artery disease prevalence using deep learning-based features. They also explored the correlation between satellite imagery and cardiometabolic diseases [15]. However, these works focus on some specific diseases without a broader understanding of the impact of the environment on diverse diseases.

#### Personalized disease risk prediction.

Predictive risk modeling predicts patients’ future disease status based on their historical medical records. Electronic health records (EHR) are generally used to provide patient medical history. As EHR data can be modeled naturally as sequential data, several deep-learning methods have been employed in previous studies for risk prediction. RETAIN [17] uses reverse time attention to capture health status from the most recent patient visits. Dipole [42] uses a bidirectional recurrent neural network to capture more complex time dependencies in EHR. In recent years, there are also transformer-based models [41, 70] and knowledge-enriched models [16, 43]. However, these models focus solely on personal patient status without utilizing information from patients’ living environments. We fill this gap by plugging our environmental embedding into patient representation according to their residence.

## 3 SatHealth Dataset

In this section, we introduce the SatHealth dataset by four components: SDoH, Environmental data, satellite imagery, and disease prevalence, as shown in Table 2. Besides data collection, we will introduce our embedding pipeline for environmental data and satellite images. We embed environmental data and satellite images across multiple geographic-level regions, including counties, ZIP Code Tabulation Areas (ZCTAs), census tracts, and Core Based Statistical Areas (CBSAs), ensuring comprehensive spatial granularity.

### 3.1 Social Determinants of Health

Social determinants of health (SDoH) refer to the environmental conditions in which people live, influencing a wide range of health outcomes and quality of life [52]. These include various socioeconomic factors, such as poverty, access to education, healthcare availability, the built environment, and community context.

#### Data Collection.

This work incorporates the Social Deprivation Index (SDI) [33] as the regional socioeconomic indicator. The SDI^3^ is a centile score comprised of seven demographic components derived from the American Community Survey (ACS): (1) the percentage of the population living below 100% of the Federal Poverty Level (FPL); (2) the percentage of individuals aged 25 years or older with less than 12 years of education; (3) the percentage of non-employed individuals aged 16–64; (4) the percentage of single-parent families with dependents under 18; (5) the percentage of households without access to a vehicle; (6) the percentage of households residing in renter-occupied units; and (7) the percentage of households living in crowded conditions.

### 3.2 Environmental Data

Environmental data contains four categories: climate, air quality, greenery, and land cover. We collect them from multiple satellite products.

#### Data Collection.

We obtained environmental variables for our dataset on Google Earth Engine (GEE) [28] following [56]. Climate variables include temperatures, humidity, solar radiation, snow cover, and wind components from ERA5-ECMWF product [48]. For air quality, we collect nitrogen dioxide (NO2) from Sentinel-5P Near Real-Time NO2 [34], total aerosols and PM2.5 from Copernicus Atmosphere Monitoring Service (CAMS) [24], and ozone from Total Ozone Mapping Spectrometer (TOMS) and Ozone Monitoring Instrument (OMI) Merged Ozone Data [2]. Greenary variables include Normalized Difference Vegetation Index (NDVI) derived from Sentinel-2 MultiSpectral Instrument (MSI) [1] and high/low vegetation greenery indices from ERA5-ECMWF. Land cover variables are area cover fractions of different land types (*e.g*., forest, water, urban) sourced from Copernicus Dynamic Land Cover products [58]. We collected all the available data in Ohio from 2016 to 2022.

#### Spatiotemporal Alignment.

Data from various satellite products have different spatial resolutions and temporal frequencies, so we align them to ensure consistency in both spatial and temporal granularity. Given a specific timestamp and an environment variable, we collect the values in various areas and obtain a heatmap, with each pixel denoting the value in a square area. We perform spatial reduction to a specific region (*e.g*., counties) by taking the average of all pixels within the region. After the reduction for every variable, we obtain an environmental vector for each specific region. We further align variables temporally by downsampling all vector series to the same monthly frequency. As land cover fractions are relatively stable, they are averaged across all timestamps to become a static variable.

#### Feature Embedding.

After the alignment process, each region has environment data as a multivariate time series. To embed the time series, we average the monthly vectors according to meteorological seasons and concatenate seasonal vectors to create annual regional embedding.

### 3.3 Satellite Images

In addition to environmental variables, satellite images offer additional visual insights into specific regions. By incorporating satellite imagery from Google Maps, we provide an implicit indicator of regional development, environmental characteristics, and land-use patterns. Compared to satellite products like Landsat 8 (30-meter resolution) and Sentinel-2 (10 to 60 meters depending on the spectral band), Google Maps provides significantly higher-resolution imagery, often better than 1 meter in many urban areas, and largely cloud-free visuals.

#### Data Collection.

We request aerial images from the Google Static Maps Application Programming Interface (API) [27]. A grid of spatial points with 500-meter spacing is created, and satellite image patches are retrieved at zoom level 17 with grid points. With the point-grid-based construction, we can establish the visual feature of an arbitrary region by aggregating satellite images within that region. This approach ensures complete coverage of Ohio, resulting in 432,918 images with a resolution of 1280×1280 pixels each, corresponding to a square area approximately 500 meters wide.

#### Feature Embedding.

We establish visual features by calculating several indices commonly used in remote sensing [10, 22, 29, 77] and computing their pixel-level statistics. For a given RGB aerial image, nine indices are calculated for each pixel based on RGB values, effectively creating new derived channels. These channels form a compound image with 12 channels alongside the original RGB channels. After that, to extract meaningful features from the compound images, we compute pixel-level statistics for each channel, including the mean, standard deviation, median, maximum, minimum, and 20-bin histogram features. This process produces feature vectors of size 300 for each satellite image. Note that images from Google Maps do not have timestamps, so the satellite image feature is static.

### 3.4 Disease Prevalence

We use regional disease prevalence as an indicator of the population health status of an area.

#### Data Collection.

We estimate disease prevalence using MarketScan [44], a real-world medical claims database. Specifically, we analyze patient encounters from the MarketScan Commercial Claims and Encounters (CCAE) database from 2016 to 2022. Each patient encounter includes a set of International Classification of Diseases (ICD-10) [12] diagnoses codes and the patient’s residency. Patient residency in MarketScan is identified by Metropolitan Statistical Areas (MSAs), a subset of CBSAs. This enables us to estimate the prevalence of each ICD-10 code for MSAs in a given year by calculating the percentage of patients associated with the code among all patients recorded in that year.

#### Data Processing.

The ICD-10 codes are organized hierarchically, allowing for a multi-level investigation of disease prevalence. For instance, the code “I10” represents essential hypertension, its parent node “I10-I1A” represents a broader group of hypertensive diseases, and the top-level code “I00-I99” encompasses circulatory system diseases. This hierarchical structure allows us to explore the prevalence of both broad disease categories and specific conditions. To capture both the overall trends and finer details, we calculated disease prevalence at the top three levels of ICD-10 codes using their first three digits, enabling a comprehensive understanding of disease patterns and correlations.

## 4 Dataset Use Cases

In this section, we first conduct a comprehensive analysis to examine the correlation between environmental data and human health status. We then demonstrate potential use cases of SatHealth through two kinds of tasks: regional public health modeling and personalized disease risk prediction. For regional public health modeling, we use regional environmental embeddings to predict the SDI score and disease prevalences, which display the power of multimodal data in modeling regional health status. For personalized disease risk prediction, we plug the environmental factors into patient representations produced by EHR risk prediction backbones according to patient residency, which shows the benefits of environmental information in predicting personal disease risks.

### 4.1 Basic Correlation Analysis

We first perform a statistical analysis to analyze the correlation between the living environment and regional disease prevalence. Note that this is an illustration of the dataset characteristics instead of a rigorous public health analysis.

#### 4.1.1 Regional Disparity.

We start by exploring the disparity in health status between regions in Ohio. For illustration, we define Columbus, Cleveland, and Cincinnati as urban areas and compare the disease occurrence with other areas in Ohio. Specifically, we calculate urban-to-rural odds ratios (OR) and their confidence intervals (CI) to show the difference in disease occurrence between the urban (Columbus, Cleveland, and Cincinnati) and other areas.

As Table 3 shows, the urban areas present a significantly higher prevalence of neonatal conditions and pregnancy-related codes. Specifically, the conditions originating in the perinatal period (P00-P96) have an odds ratio of 1.479 (95% CI: 1.418–1.542), and codes for pregnancy, childbirth, and the puerperium (O00-O9A) are also more frequent in urban areas (OR: 1.16, 95% CI: 1.131–1.190). This disparity could have originated from the higher in-hospital ratio of births [65] and limited access to maternity and prenatal care [21, 66, 72].

Moreover, rural areas have more mortality cases with ill-defined and unknown causes (ICD-10: R99, OR: 0.311, 95% CI: 0.227–0.427). This kind of coding is less informative and reflects a relatively lower data quality from the rural healthcare system [46, 68]. Besides, as the US Centers for Disease Control and Prevention (CDC) reported [11], rural residents tend to be older and sicker, and they have higher rates of cigarette smoking, high blood pressure, and obesity. Correspondingly, we observed higher prevalences of circulatory system diseases (I00-I99) as well as Endocrine, nutritional, and metabolic diseases (E00-E89). Specifically, rural areas have higher prevalences for chronic rheumatic heart diseases (OR: 0.631, 95% CI: 0.584–0.681), hypertensive diseases (OR: 0.743, 95% CI: 0.735–0.752), obesity (OR: 0.773, 95% CI: 0.761–0.785), and diabetes (OR: 0.792, 95% CI: 0.778–0.806). We show these results in Tabel 3, and more comprehensive results can be found in supplementary C. These findings help us in discovering health inequalities and support policy making [39, 71]. Furthermore, such observations and findings from public health data can help policy development, such as encouraging network development and telemedicine, and improving the rules for Medicare payments to providers [19, 55].

#### 4.1.2 Factor Correlations.

**Figure 2:**
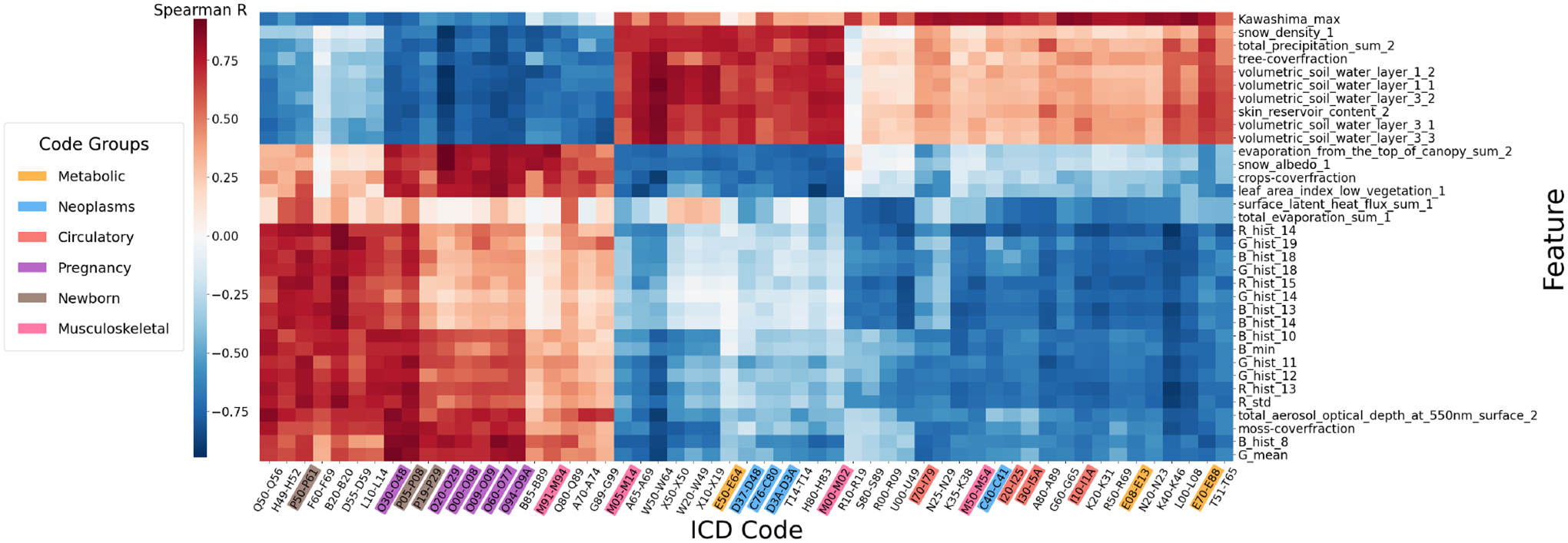
Feature correlations - second level ICD

In addition to the odds ratio, we calculate Spearman’s rank correlation coefficient to investigate the correlation between environmental factors and multi-level ICD code prevalence across spatial regions. We show the pairwise correlations between level-2 ICD codes and environmental features in Figure 2. Note that this figure only shows subsets of ICD codes and features with significant correlations. Additionally, we use color shading on ICD codes to differentiate between disease categories. More detailed results on a broader set of ICD codes are shown in the supplementary results.

We observed several outstanding color blocks in Figure 2, showing groups of diseases with distinct correlations with specific feature subsets. Firstly, a group of newborn or pregnancy-related conditions (O and P codes) with a purple or brown background lies on the left-hand side, with a significant negative correlation with tree cover fraction and volumetric soil water. Such correlation can be a reflection of the higher prevalence of newborn or pregnancy-related conditions in urban areas we found previously, as urban soil tends to be compact with lower water content [51]. In contrast, several neoplasm conditions show the opposite correlation pattern, which reflects a higher prevalence of tumors in rural areas [57]. Moreover, the correlation between neoplasms and soil water may also be explained by the higher mobility of chemical pollutants with higher precipitation and moisturized soil [8].

Moreover, some circulatory system diseases in orange color on the right-hand side, such as hypertensive diseases (I10-I1A) and heart diseases (I30-I5A). A similar pattern also applies to diabetes mellitus (E08-E13) and metabolic disorders (E70-E88). These diseases correlate positively with the Kawashima index [35] and negatively with many satellite image features. As the Kawashima index is negatively correlated to chlorophyll content [59], and image features also provide information about pixel color distribution, these correlations could be explained by the discovered benefit of green space on cardiovascular disease and diabetes [4, 5].

These results highlight the significant impact of the living environment on human health, consistent with existing studies [36, 60, 63]. They also demonstrate the potential of SatHealth to enhance AI models for health-related tasks (*e.g*., regional public health modeling and personalized disease risk prediction).

### 4.2 Regional Public Health Modeling

Regional public health modeling aims to predict health outcomes for the community or regional population. It helps identify health disparities and guide targeted interventions to improve public health outcomes and resource allocation [39, 71]. In this subsection, we use the created regional environment embeddings to predict regional SDI and disease prevalence to reveal the relationship between living environment and population health status.

We divide the environmental features into two subsets according to data structure: (1) **Dynamic environmental factors (DEnv)** refers to environmental factors that change over time, including climate, air quality, and greenery variables; (2) **Static environmental factors** refers to environmental factors that are stable over time, which include **Land Cover (LC)** and **Satellite images (Img)**. For baselines, we train random forest regressors respectively on the feature subsets to show their fundamental functional relevance. After that, we combine all modalities **(All)** and display how this helps in regional health status modeling.

#### 4.2.1 SDoH Regression.

The upper part of Table 4 presents the performance of the Social Deprivation Index (SDI) prediction. The performance metrics include Mean Absolute Error (MAE), Mean Squared Error (MSE), and *R*^2^, providing a comprehensive assessment of model accuracy and explanatory power. It is worth pointing out that we normalize the prevalence target by z-score normalization such that they have a standardized deviation of 1.

##### Overall SDI.

When predicting overall SDI, the dynamic features (DEnv) achieve the best performance, yielding the lowest MAE and highest *R*^2^. Besides, the combined feature (All) performs slightly worse but is comparable to DEnv, suggesting that DEnv variables predominantly drive the prediction. In contrast, land cover and image embeddings show suboptimal performance, likely because they capture only ground-level views, making it challenging to predict a comprehensive socioeconomic score.

**Population Characteristics** include poverty, education, and employment status. In this category, the best-performing features vary, suggesting different dependencies of SDoH on environmental factors. Specifically, the poverty ratio is better predicted by image features, likely due to their ability to capture urban-rural disparities, such as housing conditions. Education status relies more on dynamic features (*i.e*., climate, greenery, air quality). Employment benefits most from multimodal fusion as a combination of economic, infrastructure, and environmental factors influences it.

**Household Characteristics** refer to household-level factors, including single-parent family percentage, housing tenure (rent or owner-occupied), vehicle ownership, and household crowdedness. The combined feature outperforms all other features in predicting household characteristics except for the single-parent family ratio. This superiority indicates that integrating multimodal features provides a more comprehensive understanding of housing-related metrics. The combined features perform poorly in predicting the single-parent family ratio, likely because dynamic features (DEnv) tend to be independent of family structure. However, it is worth noting that none of these features are effective predictors for crowding prediction, with the best *R*^2^ score around 0.06.

#### 4.2.2 Disease Prevalence Regression.

There are thousands of diseases, but only a small subset correlates significantly with environmental features. Therefore, we focus on diseases reported to correlate with environmental factors [4, 5]: neoplasms (C00-D49), endocrine, nutritional, and metabolic diseases (E00-E89), and circulatory system diseases (I00-I99). Moreover, we explored several subcategories, including diabetes (E08-E13), metabolic disorders (E70-E88), hypertensive diseases (I10-I1A), and ischemic heart diseases (I20-I25).

##### Overall Comparison.

We show the regression performance of predicting the prevalence of the diseases in the lower part of Table 4. For most diseases (*e.g*., Neoplasms and Metabolic diseases), combining all modalities (All) achieves the best prediction performance, demonstrating the essential role of multimodal environmental factors in modeling the regional prevalence of these diseases.

##### Neoplasms.

Neoplasms (C00-D49) include all kinds of tumors, including both malignant and benign. Table 4 shows that dynamic features (DEnv) have better prediction performance than land cover and satellite, as these features contain carcinogens potentially related to some specific cancer, such as solar radiation [4] and air pollutants [64]. Moreover, by combining all features, the regression model gains 0.086 improvement in *R*^2^, the highest improvement among the explored diseases. Such improvement demonstrates that the three feature sets are complementary when modeling neoplasm prevalence.

##### Metabolic diseases.

As for endocrine, nutritional, and metabolic diseases (E00-E89), land cover (LC) and satellite image (Img) features perform consistently better than dynamic factors (DEnv). Especially for metabolic disorders (E70-E88), the *R*^2^ score on dynamic factors is 0.064 lower than the other two features. This is intuitively reasonable as these diseases are more related to our lifestyle [37, 54], and the effect of air quality or climate tends to be long-term and maybe implicit [78].

##### Circulatory system diseases.

Finally, it can be seen that the three feature modalities gain high but similar performance for circulatory system diseases (I00-I99), and the improvement of combined features is not significant. This could be due to each feature modality’s relatively high performance, making it hard to improve further. On the other hand, land cover (LC) and satellite features (Img) have higher performances than dynamic factors (DEnv) for ischemic heart diseases (I20-I25), possibly due to the reported correlation between green space and ischemic heart diseases [5].

### 4.3 Personalized Disease Risk Prediction

In this subsection, we use living environmental data to help predict patient-level disease risks. Specifically, we focus on the next visit prediction and 1-year predictive modeling tasks. We use the patient visit history data from the MarketScan database [13] to conduct experiments. We build models to predict the level-2 ICD codes within a patient visit with all previous visits from the patient. We define the multi-label ground truth in two ways: **Next visit** setting takes one visit as the ground truth and use all previous visits as input no matter how long it is from the target visit to its previous visit, while **1-Year Predictive Modeling** takes all codes within the next one year of the patient’s last known visit. In addition to the visit history, we concatenate the embedding of the patient’s living environment to the patient representation produced by the visit encoder network to enhance the backbone model. The embedding of the patient’s living environment is created by data within the patient’s residence area in the year of the patient’s last known visit.

We implemented several backbone networks for comparison. They are well-known architectures handling sequences, including LSTM [31] and Transformer [62], and models designed for medical data such as RETAIN [17] and Dipole [42]. This task can be formulated as a multi-label problem, so we use the macro average AUROC score (mAUC) across all diseases as the basic evaluation metric. Additionally, we use the macro average AUROC score of the top 10 performed diseases (mAUC-t10). Furthermore, as this model predicts the disease risk for each patient, we calculate the recall at k (Recall@k) metric to evaluate the model’s capability of producing correct disease warnings for patients.

We put the experimental results in Table 5. We highlight the best performance among the two variations in bold for each backbone model. Using environmental information boosts model performance in most cases, especially for the recall, indicating that the patient’s living environment may help us identify their disease risks. Moreover, for the next visit prediction task, models with environmental information tend to have much higher mAUC scores for top-performed diseases, which shows the capability of environmental data in differentiating patients with various disease risks.

### 4.4 Spatiotemporal Generalizability Analysis

The living environment varies in different areas and is subject to change over time. Therefore, users must be careful about spatiotemporal distribution shifts when applying models trained on SatHealth to other regions or conducting long-term forecasting. To find out how this affects the model performance with SatHealth, we conducted experiments on three cases of spatiotemporal generalization on regional public health modeling. Moreover, we designed a spatiotemporal-enhanced regression strategy to improve model robustness. In this subsection, we show the experimental performance of the enhanced model in the three generalization scenarios: spatial interpolation (*e.g*., missing value imputation [6]), spatial extrapolation (*e.g*., distant region generalization [45, 53]), and temporal forecasting [30, 50]. We provide more experimental details in the supplementary Section C.

**Figure 3:**
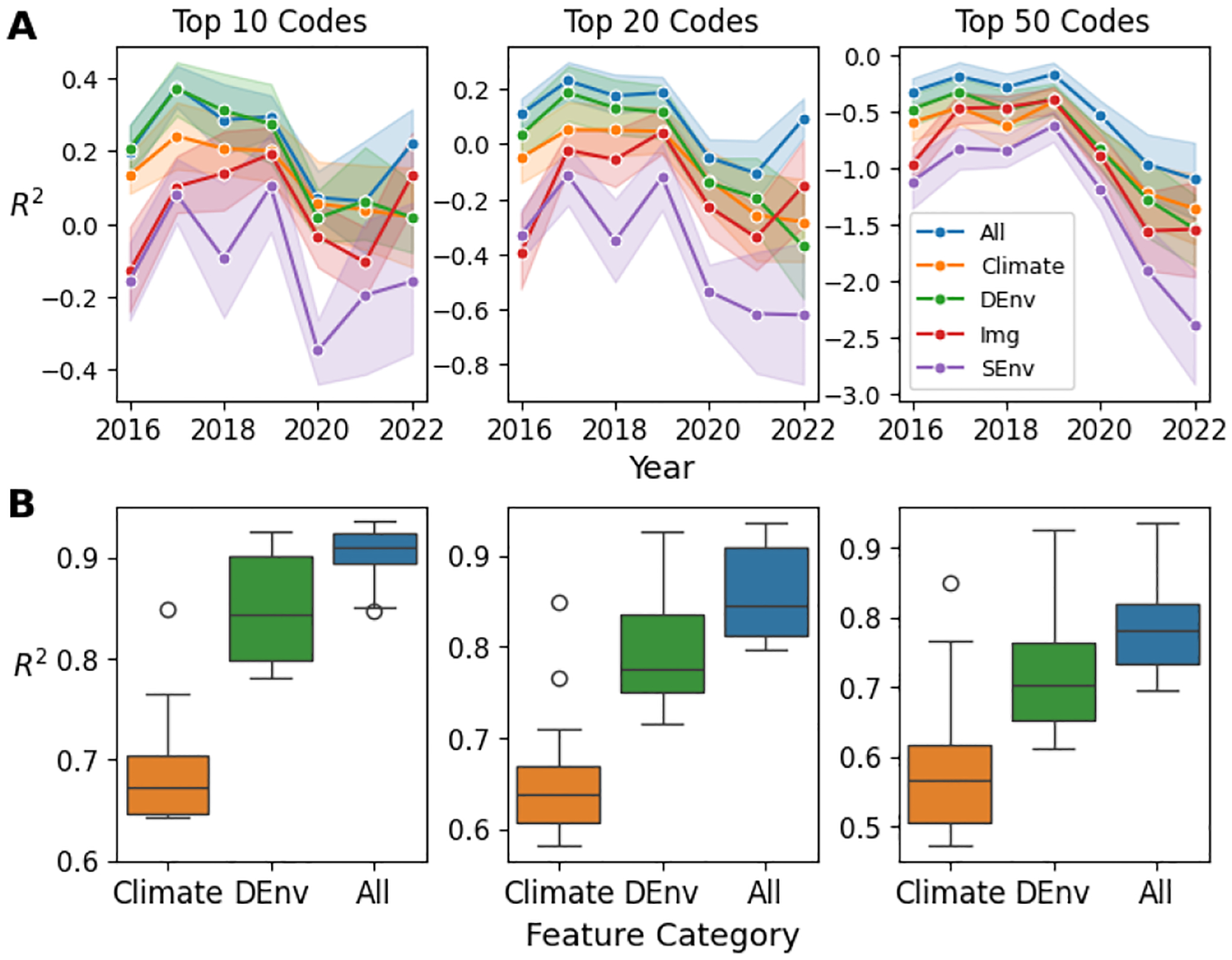
Random Forest Regression Performance from Different Feature Groups. (A) *R*^2^ score per year for spatial interpolation. (B) *R*^2^ score for temporal extrapolation.

We start by evaluating the generalizability of basic regression models without spatiotemporal enhancements. Using *R*^2^ scores, we show the top 10, 20, and 50 performing diseases. Figure 3 compares model performance across input modalities for (A) spatial interpolation and (B) temporal forecasting. LC and Img models are excluded from temporal forecasting due to their time independence. A model trained solely on climate variables is also included for comparison. For spatial interpolation, we stratify results by year. As shown in Figure 3B, Combined features show higher stability to temporal shift, likely due to reduced feature variance over time. Additionally, the combined features improve spatial generalization. Dynamic features, including climate, greenery, and air quality, exhibit stronger spatial robustness, possibly due to their pronounced spatial clustering effect.

Next, we show how spatiotemporal information can improve model generalizability. We train boosting models as described in Section C. Models with neighborhood information are annotated with “+S”, and models with history information are annotated with “+T”. We compared the combined feature model (All) with the Dynamic feature model (DEnv) under three generalization cases, and the result is shown in Table 6. We calculate the average for SDoH variables and the top-10 performing ICD codes. It can be seen that incorporating spatiotemporal information can be beneficial to model robustness, as the “+T+S” models are either the best or the second best for all cases. Although not the best, the “All+T+S” model is comparable to the best, providing a general solution that works under all cases. Moreover, the combined feature models tend to perform better than the DEnv models, further showing the multimodal models’ benefits. Finally, we find that our model performs poorly when predicting SDoH under spatial extrapolation, which may indicate a high spatial disparity between regions, making the spatial generalization of the SDoH model challenging.

## 5 Web Application Deployment

**Figure 4:**
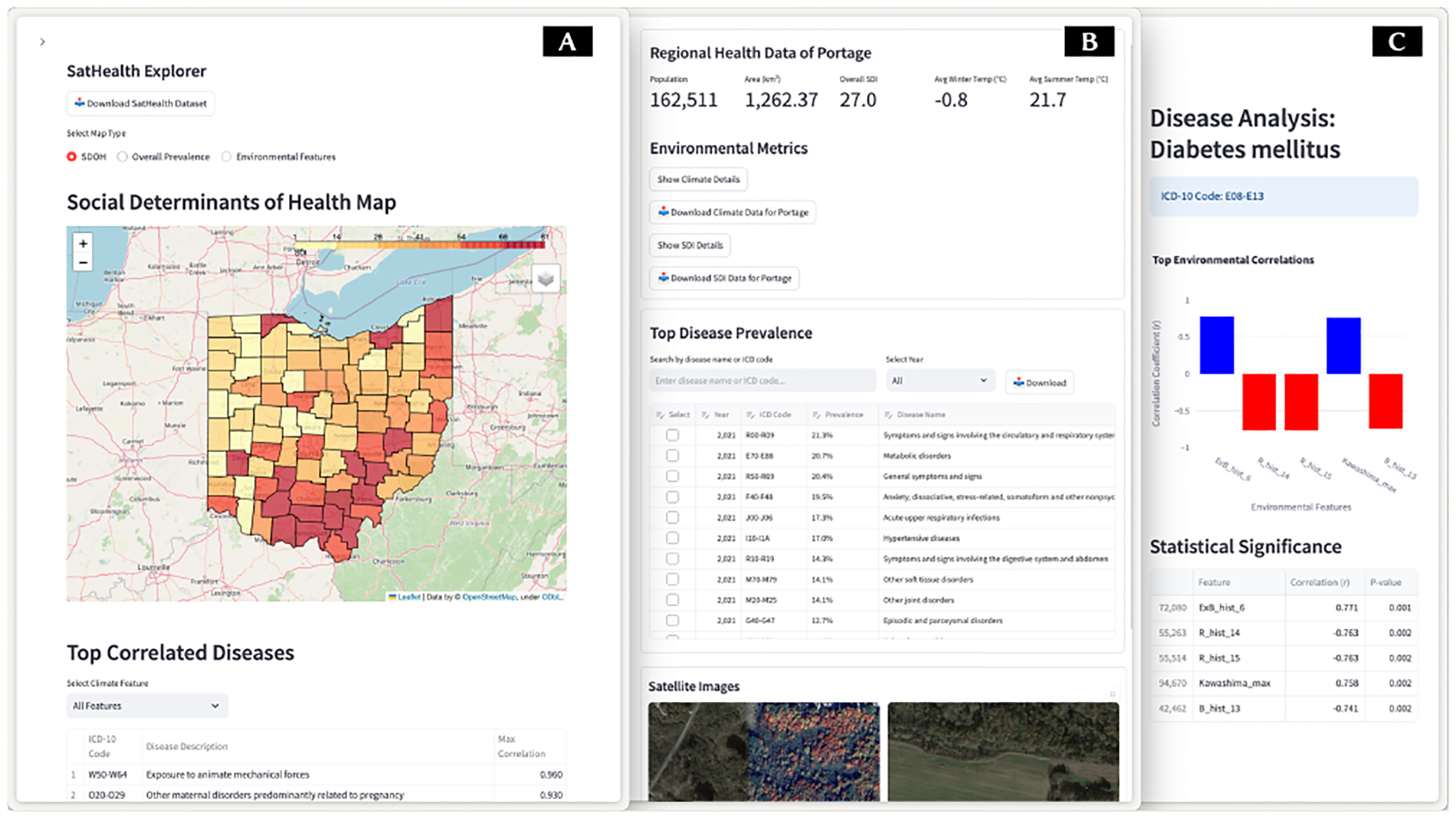
UI design of the proposed dataset

To improve the utility of our dataset, we designed a web-based application to explore and access SatHealth. The system design is shown in Figure 4.

The default page (Figure 4A) is the satellite map of Ohio, with the overall SDI score for each county shown in the heatmap. Users can select different variables to be displayed in the heatmap, including SDI, environmental variables, and disease prevalence of the user-selected ICD code. For time-dependent variables, there’s a slide bar for users to define the time range of the data shown. Moreover, diseases with a high correlation to environmental features are shown below the map; users can also select specific environmental features to check their correlation to the map. We also provide the button to download the whole dataset at the top of this page. More information about data access can be found in Section A.4.

Once users click on one of the regions in the map, its basic information will be shown in a new column on the right-hand side (Figure 4B). There will be three sub-panels in this sidebar. On the top, basic metrics, including population and total area of the region, are shown, together with the overall SDI score and seasonal average temperature. When users click the “Show Details” button, a drop-down list will appear to display more variables. Buttons are also provided to download the SatHealth subset in the selected region. In the middle, there’s a list of the most prevalent diseases in the selected region; users can also search for ICD codes to see their prevalence. At the bottom of this sidebar, random satellite images in the selected region will be displayed.

Users can check the selection box in the disease list. The right-hand side column will be updated (Figure 4C) to show a bar chart of the correlation score of the selected disease with the top 5 correlated environmental features in a bar chart. The correlation score and corresponding p-value will also be displayed below the bar chart. Users can search for a specific variable to check its correlation to the current disease.

## 6 Limitations and Future Work

Our work still has several limitations. First, SatHealth is currently restricted to Ohio due to budget constraints, but we will gradually expand our dataset to include more states, toward full US coverage. Furthermore, although environmental data showed effectiveness in our experiment, our embedding method of such multimodal data is relatively straightforward using feature engineering. We will explore more complicated deep-learning strategies to unlock the power of multimodal environmental data. Finally, the individual residences in our dataset are coarse due to privacy issues, making the living environment less representative. Despite these limitations, we believe SatHealth still provides a solid foundation and a good starting point for research on the interplay between the living environment and human health.

## 7 Conclusion

In this study, we developed SatHealth, a multimodal public dataset in Ohio consisting of environmental variables, satellite images, SDoH, and regional disease prevalence. To the best of our knowledge, SatHealth is the first dataset in the US that combines regional environmental characteristics with a healthcare database. We designed an embedding pipeline to fuse multimodal environmental data and produce regional living environment representations. Using the representation, we conduct experiments on two tasks: regional public health modeling and personalized disease risk prediction. The experimental results show that multimodal environmental information helps boost model performance in these problems. We also find that leveraging spatiotemporal information helps improve model robustness. Finally, we deployed a web application for users to explore and download our dataset.

## Supplementary Material

1

## Figures and Tables

**Figure 1:**
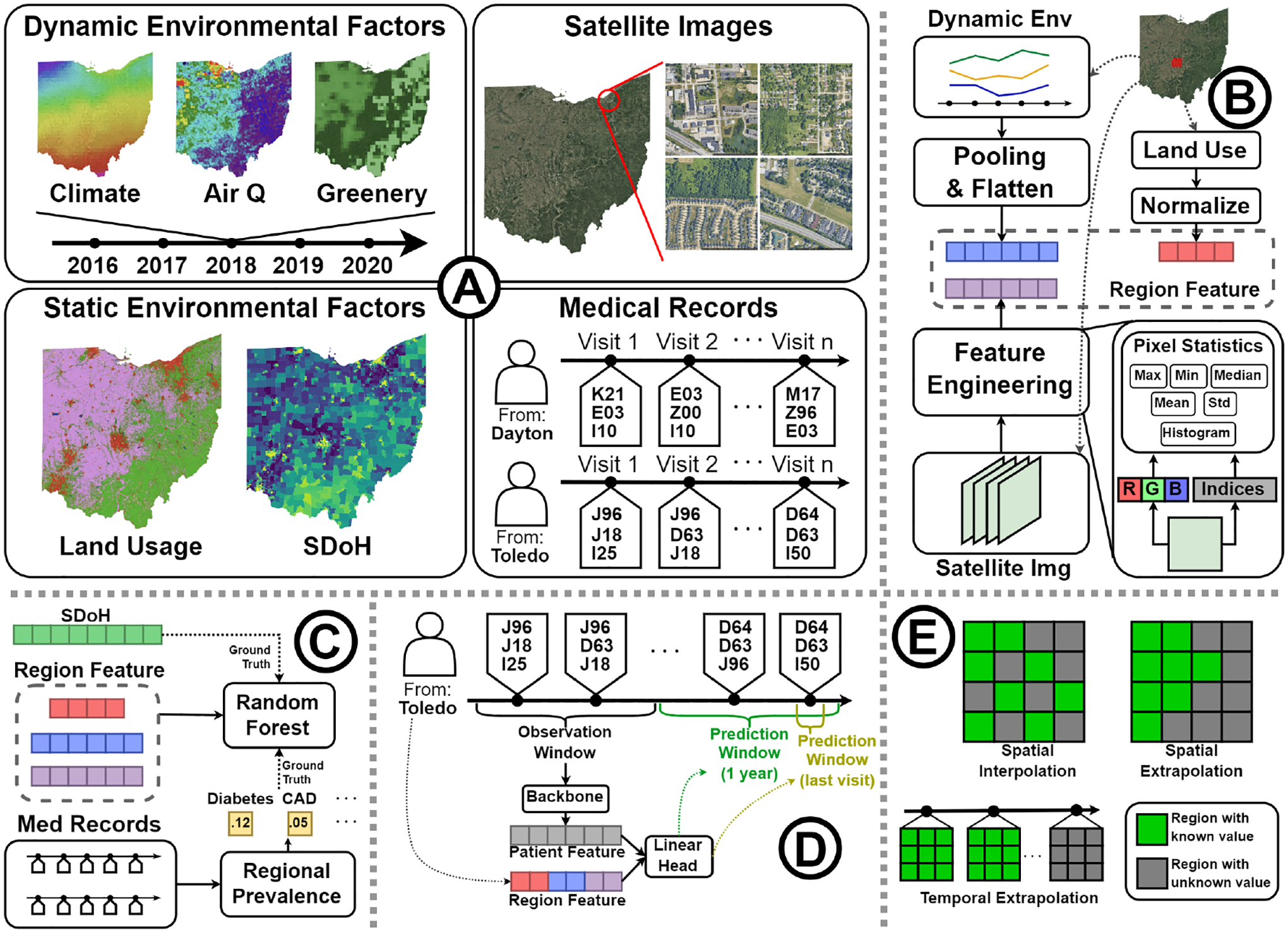
Overview of SatHealth and Experimental framework. (A) The components of SatHealth. (B) The regional embedding pipeline. We use all dynamic factors, satellite images, and land usage as the multimodal environmental features of a region. (C) We use the regional embedding to predict the prevalence of all kinds of diseases and SDoH within that region. (D) We combine patient representation with the regional embedding of patient residence to predict patient disease risk. (E) We test model robustness on three generalization scenarios.

**Table 1:** A comparison of SatHealth to related datasets combining environmental and health data

Dataset	Location	Target	Target data source	Imagery	Year span	Public
Barboza et al.	31 European countries	Mortality	Eurostat	✘	2015	✘
Temenos et al.	8 European cities	COVID-19	OWD	✘	2020–2021	✘
SatelliteBench	81 municipalities in Colombia	Dengue outbreak, Poverty, Access to school	SIVIGILA, Census	Sentinel-2	2016–2018	✓
SustainBench	Global	BMI, child mortality,water quality,sanitation	Surveys	LandSat, Street view	1996–2019	✓
MedSat	England	Medical prescription of 7 conditions	NHS	Sentinel-2	2019–2020	✓
**SatHealth**	Ohio, US	SDoH, All ICD code prevalence	SDI, MarketScan	Google Maps	2016–2022	✓

OWD: Our World in Data platform; NHS: National Health Services; SIVIGILA: Colombian Public Health Surveillance System

**Table 2:** The number of variables in each category

	Modality	Category	Statistics
SDoH	Tabular	SDI	8 Variables
Environmental	Tabular	Land Cover	9 Variables
	Time Series (Monthly)	Climate	28 Variables, 7 years
		Air Quality	4 Variables, 7 years
		Greenery	4 Variables, 7 years
Satellite Imagery	Image	-	432918 Images
			3 Channels (RGB)
			9 Calculated Indices
Medical Records	Time Series (Yearly)	ICD code Prevalence	1377 unique codes
			7 years coverage
			2141777 patients

**Table 3:** ICD codes with top Urban-rural relevance by Odds Ratio

Category	Code	Odds Ratio	95% CI	Description
Urban Prevalent	P00-P96	1.479	(1.418, 1.542)	Certain conditions originating in the perinatal period
	O00-O9A	1.160	(1.131, 1.190)	Pregnancy, childbirth and the puerperium
Rural Prevalent	R99	0.312	(0.227, 0.427)	Ill-defined and unknown cause of mortality
	I00-I99	0.765	(0.756, 0.773)	Diseases of the circulatory system
	I05-I09	0.631	(0.584, 0.681)	Chronic rheumatic heart diseases
	I26-I28	0.735	(0.687, 0.786)	Pulmonary heart disease and diseases of pulmonary circulation
	I60-I69	0.736	(0.701, 0.772)	Cerebrovascular diseases
	I10-I1A	0.743	(0.735, 0.752)	Hypertensive diseases
	E00-E89	0.785	(0.777, 0.793)	Endocrine, nutritional and metabolic diseases
	E40-E46	0.636	(0.583, 0.694)	Malnutrition
	E70-E88	0.737	(0.728, 0.745)	Metabolic disorders
	E65-E68	0.773	(0.761, 0.785)	Overweight, obesity and other hyperalimentation
	E08-E13	0.792	(0.778, 0.806)	Diabetes mellitus

**Table 4:** Regression performance for SDI components and disease prevalence

		MAE ↓				MSE ↓				R^2^ ↑			
Category	Target	DEnv	LC	Img	All	DEnv	LC	Img	All	DEnv	LC	Img	All
SDoH	SDI	**0.564**	0.621	0.618	**0.564**	**0.461**	0.590	0.544	0.464	**0.558**	0.434	0.478	0.555
	- pct_Poverty_LT100	0.516	**0.473**	0.480	**0.473**	0.567	0.424	**0.403**	0.413	0.277	0.458	**0.486**	0.473
	- pct_Education_LT12years	**0.495**	0.600	0.572	0.536	**0.526**	0.762	0.606	0.552	**0.343**	0.048	0.243	0.311
	- pct_NonEmployed	0.533	0.593	0.560	**0.532**	0.740	0.679	0.659	**0.577**	0.140	0.210	0.234	**0.329**
	- pct_Single_Parent_Fam	0.509	0.443	**0.414**	0.433	0.680	0.424	**0.369**	0.396	−0.285	0.199	**0.302**	0.251
	- pctHH_No_Vehicle	0.484	0.512	0.519	**0.438**	0.709	0.743	0.675	**0.562**	0.406	0.377	0.434	**0.529**
	- pctHH_Renter_Occupied	0.445	0.462	0.447	**0.406**	0.382	0.444	0.366	**0.315**	0.580	0.513	0.598	**0.654**
	- pctHH_Crowding	0.682	0.709	0.697	**0.675**	1.113	1.222	1.192	**1.052**	0.009	−0.089	−0.063	**0.062**
Disease Prevalence	Neoplasms	0.389	0.392	0.391	**0.327**	0.284	0.288	0.284	**0.215**	0.657	0.621	0.632	**0.743**
	Metabolic diseases	0.424	0.384	0.385	**0.366**	0.299	0.248	0.250	**0.228**	0.686	0.706	0.704	**0.742**
	- Diabetes mellitus	0.295	0.268	0.271	**0.267**	0.158	0.137	0.139	**0.127**	0.831	0.848	0.845	**0.861**
	- Metabolic disorders	0.326	**0.249**	0.250	0.284	0.195	0.116	**0.115**	0.142	0.781	0.845	**0.846**	0.827
	Circulatory system diseases	0.227	0.205	**0.200**	0.212	0.086	0.075	**0.072**	0.078	0.900	0.909	**0.912**	0.909
	- Hypertensive diseases	0.198	0.194	0.186	**0.177**	0.063	0.062	0.058	**0.052**	0.928	0.927	0.930	**0.939**
	- Ischemic heart diseases	0.304	**0.249**	0.250	0.254	0.185	0.123	**0.122**	0.142	0.815	0.873	**0.874**	0.855

All: embeddings by combining all modalities; “-” denotes subcategories

**Table 5:** Prediction performance of personalized disease risk prediction

		Next Visit Prediction					1-Year Diagnosis Code Prediction				
		mAUC↑	mAUC-t10↑	Recall@5↑	Recall@10↑	Recall@50↑	mAUC↑	mAUC-t10↑	Recall@5↑	Recall@10↑	Recall@50↑
LSTM	EHR	0.481	0.745	0.240	0.253	0.416	0.487	0.770	**0.503**	**0.537**	**0.687**
	+Env	**0.538**	**0.767**	**0.288**	**0.325**	**0.561**	**0.551**	**0.815**	0.489	0.508	0.642
RETAIN	EHR	0.476	0.678	**0.273**	**0.301**	**0.456**	0.492	0.703	0.198	0.221	0.463
	+Env	**0.551**	**0.816**	**0.273**	0.287	0.445	**0.543**	**0.764**	**0.205**	**0.265**	**0.503**
Dipole	EHR	0.600	0.869	0.263	0.302	0.487	0.660	0.929	0.477	0.504	0.641
	+Env	**0.722**	**0.942**	**0.416**	**0.480**	**0.724**	**0.734**	**0.932**	**0.553**	**0.587**	**0.769**
Transformer	EHR	0.484	0.679	0.215	0.240	0.439	0.503	0.706	0.310	0.333	0.523
	+Env	**0.549**	**0.738**	**0.234**	**0.265**	**0.495**	**0.553**	**0.732**	**0.341**	**0.377**	**0.584**

**Table 6:** Model *R*^2^ scores for Spatiotemporal Generalization. Bold: best results, underlined: second best.

	Spa. In.		Spa. Ex.		Temp. For.
Features	SDoH	Prev.	SDoH	Prev.	Prev.
DEnv	0.183	0.667	−0.186	0.552	0.811
DEnv + T	0.161	0.665	−0.301	0.522	0.868
DEnv + S	0.183	0.705	−0.261	0.552	0.759
DEnv + T + S	0.165	**0.711**	−0.296	**0.624**	0.820
All	0.244	0.665	**0.035**	0.543	0.902
All + T	0.238	0.671	−0.005	0.525	**0.928**
All + S	**0.275**	0.699	0.014	0.572	0.879
All + T + S	0.265	0.709	0.026	0.619	0.914

Prev.: Disease Prevalence; Spa.: Spatial; Temp.: Temporal; Ex.: extrapolation; In.: interpolation; For.: forecasting

## CCS Concepts

• **Applied computing** → *Health informatics*; • **Social and professional topics** → *Medical records*; • **Computing methodologies** → *Learning latent representations*.

## Footnotes

1
https://aimed-sathealth.net

2
https://github.com/Wang-Yuanlong/SatHealth

3
https://www.graham-center.org/maps-data-tools/social-deprivation-index.html
