# Supplementary material for: SatHealth: A Multimodal Public Health Dataset with Satellite-based Environmental Factors
Source: KDD. Author manuscript; Available in PMC 2025 Aug 12. (PMC12340727; doi:10.1145/3711896.3737440)
Supplement: 1 [file NIHMS2102035-supplement-1.pdf]

A Dataset Information

Table 7: SatHealth Data Scope

	Spatial Range	Time Span
SatHealth	Ohio	2016-2022
	Spatial Res.	Temporal Res.
Climate/Air Q/Greenery	Cty/ZCTA/CT/CBSA	Month
Land cover	Cty/ZCTA/CT/CBSA	N/A
SDoH (SDI score)	Cty/ZCTA/CT	Year
Disease Prevalence	CBSA	Year
Satellite image	500m×500m per image	N/A

Res.: Resolution; Air Q: Air quality; CBSA: Core Based Statistical Area
Cty: County; ZCTA: Zip Code Tabulation Area; CT: Census Tract

A.1 Data Scope

We started with data curation in Ohio while participating in the Ohio O-SUDDEn program [23]. We noticed the shortage of medical datasets with environmental health risk factors. Therefore, we develop SatHealth, a multimodal public health dataset with environmental factors in multiple categories. We use the data from 2016 to 2022 that is available for all modalities.

We show the scope of SatHealth in Table 7. SatHealth includes monthly aggregated data for climate, air quality, and greenery index at multiple geographical area levels, including counties, ZIP Code Tabulation Areas (ZCTAs), census tracts, and Core Based Statistical Areas (CBSAs). Land cover data in SatHealth is time-independent and denotes the area fraction of multiple land usage types at multiple geographical area levels mentioned above. Moreover, our dataset contains the latest aerial satellite images requested from Google Maps [27]; each image covers a square area around 500m wide. We've paid around 2500$ (including free credits) to extract satellite images from the Google Maps API.

For health-related outcomes, we have the Social Deprivation Index (SDI) in 2019. We also provide yearly prevalences for all diseases identified by ICD codes estimated from medical claims in Ohio extracted from the MarketScan database [44]. We also paid for authorized access to the MarketScan database.

Although SatHealth was created in Ohio using data from 2016-2022, our framework is scalable, and we will gradually cover the whole US and keep updating new data after 2022. We also provide the code for users to create their customer data.

A.2 Ethics and Fairness Statement

We adhere to the data license and usage agreement of all data sources when constructing SatHealth. The list of licenses and agreements can be found in our arXiv version.

The MarketScan database used in this research is fully Health HIPAA compliant, de-identified, and has very minimal risk of the potential for loss of privacy. We will only publish regional statistics from the original medical claims and exclude regions with fewer than 10 people. Thus, there's a minimal privacy leakage issue for our medical data.

A.3 Data License

The dataset is released under the CC BY-SA 4.0 license.

A.4 Data Access

Users can explore and download our dataset at https://aimed-sathealth.net with documentation at https://github.com/Wang-Yuanlong/SatHealth. We provide monthly data for climate, air quality, and greenery of multiple cartographic levels mentioned above for users to download. We also offer regional SDI scores and disease prevalence for all ICD codes in the MarketScan database. However, personalized data is not available. Moreover, the collection of satellite images from Google Maps is not available due to copyright concerns, but we provide scripts and a manual for users to extract them from the API Google provided.

B Dataset Details

This section contains the description of the file structure in which we publish the SatHealth data, and the description of the data sources of SatHealth.

Due to the page limitation, please refer to our arXiv version for the full supplementary.

C Additional Experimental Details and Results

In this section, we provide experimental details of our spatiotemporal generalizability analysis. Moreover, additional experimental results for regional disparity and feature correlations are provided.

Due to the page limitation, please refer to our arXiv version for the full supplementary.
